# *p*-Tolyl bis­(cyclo­hexyl­amido)­phosphinate

**DOI:** 10.1107/S1600536811029722

**Published:** 2011-07-30

**Authors:** Akbar Raissi Shabari, Mehrdad Pourayoubi, Afsaneh Taghizadeh, Farnaz Ghoreishi, Banafsheh Vahdani

**Affiliations:** a Faculty of Chemistry, North Tehran Branch, Islamic Azad University, Tehran, Iran; b Department of Chemistry, Ferdowsi University of Mashhad, Mashhad 91779, Iran

## Abstract

The P atom in the title mol­ecule, C_19_H_31_N_2_O_2_P, is in a distorted tetra­hedral configuration with the bond angles in the range 101.48 (10)–118.58 (9)°. The N—H units have a *syn* orientation with respect to one another. In the crystal, mol­ecules are connected *via* two different inter­molecular N—H⋯O(P) hydrogen bonds into chains along the *a* axis in which the O atom of the P=O group acts as a double acceptor.

## Related literature

For background to phospho­ramidate compounds, see: Pourayoubi *et al.* (2011 ▶). For bond lengths in related structures, see: Sabbaghi *et al.* (2011 ▶); Rudd *et al.* (1996 ▶). For double hydrogen-bond acceptors, see: Steiner (2002 ▶).
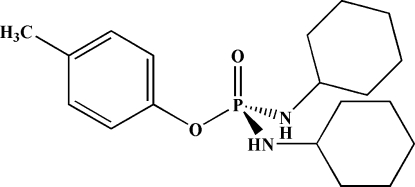


## Experimental

### 

#### Crystal data

C_19_H_31_N_2_O_2_P
*M* *_r_* = 350.43
Monoclinic, 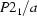

*a* = 9.131 (5) Å
*b* = 19.333 (5) Å
*c* = 11.291 (5) Å
β = 99.247 (5)°
*V* = 1967.3 (15) Å^3^
*Z* = 4
Mo *K*α radiation
μ = 0.15 mm^−1^
*T* = 291 K
0.38 × 0.12 × 0.08 mm

#### Data collection

Stoe IPDS II image plate diffractometer
Absorption correction: multi-scan [*MULABS* (Blessing, 1995 ▶) in *PLATON* (Spek, 2009 ▶)] *T*_min_ = 0.992, *T*_max_ = 1.000
14835 measured reflections
5296 independent reflections
1925 reflections with *I* > 2σ(*I*)
*R*_int_ = 0.091

#### Refinement

*R*[*F* ^2^ > 2σ(*F* ^2^)] = 0.052
*wR*(*F* ^2^) = 0.090
*S* = 0.76
5296 reflections
218 parameters
H-atom parameters constrained
Δρ_max_ = 0.21 e Å^−3^
Δρ_min_ = −0.26 e Å^−3^

### 

Data collection: *X-AREA* (Stoe & Cie, 2005 ▶); cell refinement: *X-AREA*; data reduction: *X-AREA*; program(s) used to solve structure: *SHELXTL* (Sheldrick, 2008 ▶); program(s) used to refine structure: *SHELXTL*; molecular graphics: *SHELXTL*; software used to prepare material for publication: *SHELXTL*, *PLATON* (Spek, 2009 ▶) and *enCIFer* (Allen *et al.*, 2004 ▶).

## Supplementary Material

Crystal structure: contains datablock(s) I, global. DOI: 10.1107/S1600536811029722/lh5287sup1.cif

Structure factors: contains datablock(s) I. DOI: 10.1107/S1600536811029722/lh5287Isup2.hkl

Supplementary material file. DOI: 10.1107/S1600536811029722/lh5287Isup3.cml

Additional supplementary materials:  crystallographic information; 3D view; checkCIF report

## Figures and Tables

**Table 1:** Hydrogen-bond geometry (Å, °)

*D*—H⋯*A*	*D*—H	H⋯*A*	*D*⋯*A*	*D*—H⋯*A*
N1—H1⋯O1^i^	0.76	2.23	2.969 (3)	163
N2—H2⋯O1^i^	0.75	2.25	2.975 (3)	162

Symmetry code: (i) 
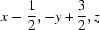
.

**Fig. 1.**
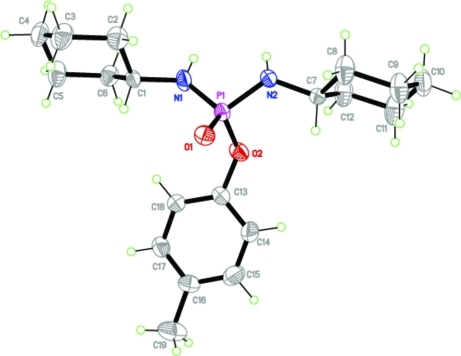
The molecular structure of the title compound with ellipsoids shown at the 50% probability level.

**Fig. 2.**
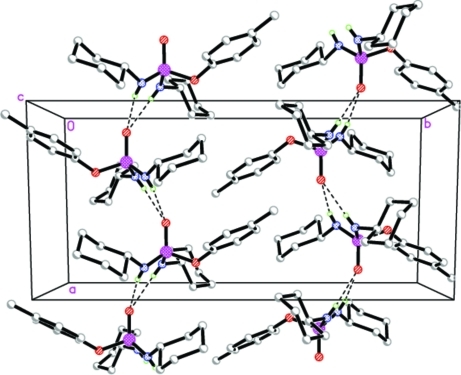
A view of the crystal packing showing the formation of chains through N—H···O hydrogen bonds (shown as dashed lines). H atoms not involved in hydrogen bonding have been omitted for clarity.

## supplementary crystallographic information

### Comment

The structure determination of title compound was performed as a part of a project in our laboratory on the synthesis of new phosphoramidate compounds (Pourayoubi *et al.*, 2011).

The P═O (1.4670 (17) Å), P—O (1.6105 (17) Å) and P—N (1.6131 (19) Å and 1.6152 (18) Å) bond lengths and the C—O—P (123.52 (15)°) and C—N—P (125.28 (16)° and 123.76 (16)°) angles match those found for other compounds with the [(N)(N)P(O)(O)] skeleton (Sabbaghi *et al.*, 2011).

The tetrahedral configuration of phosphorus atom is significantly distorted (Fig. 1) as it has been noted for other phosphoramides and their chalco-derivatives (Rudd *et al.*, 1996); the bond angles at the P atom vary in the range from 101.48 (10)° [for O2—P1—N2 angle] to 118.58 (9)° [for O1—P1—N2 angle]. Cyclohexyl groups are in a chair conformation with the adjacent NH groups oriented equatorially.

The O atom of the P═O group acts as a double hydrogen-bond acceptor (Steiner, 2002). In the crystal structure, each molecule is hydrogen-bonded to two adjacent molecules through [N—H]_2_···O(P) hydrogen bonds forming linear chains parallel to [100] (Fig. 2, Table 1).

### Experimental

Synthesis of 4-CH_3_–C_6_H_4_–O–P(O)Cl_2_ 4-CH_3_–C_6_H_4_–O–H (0.18 mol) and P(O)Cl_3_ (0.54 mol) were refluxed for 8 h and afterwards the excess of P(O)Cl_3_ was removed in vacuum.

Synthesis of title compound To a solution of 4-CH_3_–C_6_H_4_–O–P(O)Cl_2_ (2 mmol) in chloroform (20 ml), a solution of cyclohexylamine (8 mmol) in chloroform (15 ml) was added at 273 K. After 4 h of stirring, the solvent was evaporated in vacuum. The solid was washed with distilled water, and then re-crystallized from a mixture of CHCl_3_/n-C_7_H_16_. Single crystals, suitable for crystallography, were obtained from a solution of the title compound in acetonitrile after slow evaporation at room temperature.

### Refinement

Though the H-atoms were visible in difference fourier maps they were included in geometrically idealized positions with C—H distances = 0.93, 0.96, 0.97 and 0.98 Å for aryl, methyl, methylene and methine type H-atoms, respectively and N-H = 0.75-0.76Å. The H-atoms were assigned *U*_iso_ = 1.5 times *U*_eq_ methyl C atom and 1.2 times *U*_eq_ of the rest of the parent atoms (C/N).

### Crystal data

**Table:**

C_19_H_31_N_2_O_2_P	*F*(000) = 760
*M**_r_* = 350.43	*D*_x_ = 1.183 Mg m^−^^3^
Monoclinic, *P*2_1_/*a*	Mo *K*α radiation, λ = 0.71069 Å
Hall symbol: -P 2yab	Cell parameters from 3912 reflections
*a* = 9.131 (5) Å	θ = 1.8–29.5°
*b* = 19.333 (5) Å	µ = 0.15 mm^−^^1^
*c* = 11.291 (5) Å	*T* = 291 K
β = 99.247 (5)°	Block, colourless
*V* = 1967.3 (15) Å^3^	0.38 × 0.12 × 0.08 mm
*Z* = 4	

### Data collection

**Table:**

Stoe IPDS II image plate diffractometer	5296 independent reflections
Radiation source: fine-focus sealed tube	1925 reflections with *I* > 2σ(*I*)
graphite	*R*_int_ = 0.091
Detector resolution: 0.15 mm pixels mm^-1^	θ_max_ = 29.2°, θ_min_ = 1.8°
ω scans	*h* = −12→9
Absorption correction: multi-scan [*MULABS* (Blessing, 1995) in *PLATON* (Spek, 2009)]	*k* = −22→26
*T*_min_ = 0.992, *T*_max_ = 1.000	*l* = −15→15
14835 measured reflections	

### Refinement

**Table:**

Refinement on *F*^2^	Primary atom site location: structure-invariant direct methods
Least-squares matrix: full	Secondary atom site location: difference Fourier map
*R*[*F*^2^ > 2σ(*F*^2^)] = 0.052	Hydrogen site location: inferred from neighbouring sites
*wR*(*F*^2^) = 0.090	H-atom parameters constrained
*S* = 0.76	*w* = 1/[σ^2^(*F*_o_^2^) + (0.0255*P*)^2^] where *P* = (*F*_o_^2^ + 2*F*_c_^2^)/3
5296 reflections	(Δ/σ)_max_ = 0.001
218 parameters	Δρ_max_ = 0.21 e Å^−^^3^
0 restraints	Δρ_min_ = −0.26 e Å^−^^3^

### Special details

**Table:**

Geometry. All e.s.d.'s (except the e.s.d. in the dihedral angle between two l.s. planes) are estimated using the full covariance matrix. The cell e.s.d.'s are taken into account individually in the estimation of e.s.d.'s in distances, angles and torsion angles; correlations between e.s.d.'s in cell parameters are only used when they are defined by crystal symmetry. An approximate (isotropic) treatment of cell e.s.d.'s is used for estimating e.s.d.'s involving l.s. planes.
Refinement. Refinement of *F*^2^ against ALL reflections. The weighted *R*-factor *wR* and goodness of fit *S* are based on *F*^2^, conventional *R*-factors *R* are based on *F*, with *F* set to zero for negative *F*^2^. The threshold expression of *F*^2^ > σ(*F*^2^) is used only for calculating *R*-factors(gt) *etc*. and is not relevant to the choice of reflections for refinement. *R*-factors based on *F*^2^ are statistically about twice as large as those based on *F*, and *R*- factors based on ALL data will be even larger.

### Fractional atomic coordinates and isotropic or equivalent isotropic displacement parameters (Å^2^)

**Table:**

	*x*	*y*	*z*	*U*_iso_*/*U*_eq_	
P1	0.71746 (8)	0.80003 (4)	0.50141 (5)	0.03970 (16)	
O1	0.87988 (16)	0.79943 (9)	0.51391 (11)	0.0481 (4)	
O2	0.66002 (18)	0.87739 (8)	0.46763 (13)	0.0530 (5)	
N1	0.6357 (2)	0.74705 (11)	0.40180 (15)	0.0540 (6)	
H1	0.5695	0.7286	0.4204	0.065*	
N2	0.6418 (2)	0.78465 (10)	0.61857 (13)	0.0488 (6)	
H2	0.5753	0.7613	0.6076	0.059*	
C1	0.7075 (3)	0.71015 (13)	0.31259 (16)	0.0433 (6)	
H1A	0.7944	0.7370	0.2994	0.052*	
C2	0.7592 (3)	0.63929 (14)	0.3529 (2)	0.0637 (8)	
H2A	0.6753	0.6125	0.3700	0.076*	
H2B	0.8301	0.6429	0.4264	0.076*	
C3	0.8310 (4)	0.60189 (16)	0.2589 (2)	0.0779 (9)	
H3A	0.8596	0.5557	0.2870	0.093*	
H3B	0.9199	0.6264	0.2465	0.093*	
C4	0.7244 (3)	0.59729 (16)	0.1408 (2)	0.0749 (9)	
H4A	0.7738	0.5756	0.0806	0.090*	
H4B	0.6397	0.5690	0.1513	0.090*	
C5	0.6727 (4)	0.66822 (17)	0.0996 (2)	0.0755 (9)	
H5A	0.6010	0.6644	0.0266	0.091*	
H5B	0.7565	0.6947	0.0813	0.091*	
C6	0.6017 (3)	0.70671 (14)	0.19495 (17)	0.0579 (7)	
H6A	0.5753	0.7532	0.1672	0.070*	
H6B	0.5116	0.6831	0.2070	0.070*	
C7	0.6472 (3)	0.83281 (12)	0.71992 (17)	0.0434 (6)	
H7A	0.7147	0.8706	0.7074	0.052*	
C8	0.7099 (3)	0.79764 (16)	0.83611 (18)	0.0739 (9)	
H8A	0.8094	0.7815	0.8318	0.089*	
H8B	0.6494	0.7577	0.8473	0.089*	
C9	0.7148 (4)	0.84609 (17)	0.94366 (19)	0.0768 (10)	
H9A	0.7512	0.8210	1.0168	0.092*	
H9B	0.7830	0.8838	0.9366	0.092*	
C10	0.5655 (4)	0.87468 (17)	0.9507 (2)	0.0772 (10)	
H10A	0.5721	0.9065	1.0178	0.093*	
H10B	0.4990	0.8374	0.9642	0.093*	
C11	0.5044 (4)	0.91182 (17)	0.8365 (2)	0.0913 (11)	
H11A	0.5660	0.9517	0.8271	0.110*	
H11B	0.4051	0.9282	0.8410	0.110*	
C12	0.4996 (3)	0.86385 (16)	0.7272 (2)	0.0715 (9)	
H12A	0.4285	0.8271	0.7321	0.086*	
H12B	0.4662	0.8900	0.6545	0.086*	
C13	0.7349 (3)	0.92389 (13)	0.40431 (19)	0.0448 (6)	
C14	0.7785 (4)	0.98514 (16)	0.4565 (2)	0.0709 (9)	
H14A	0.7614	0.9946	0.5338	0.085*	
C15	0.8477 (4)	1.03306 (16)	0.3956 (2)	0.0788 (10)	
H15A	0.8768	1.0749	0.4327	0.095*	
C16	0.8753 (3)	1.02153 (15)	0.2822 (2)	0.0572 (7)	
C17	0.8292 (3)	0.95964 (15)	0.2303 (2)	0.0596 (8)	
H17A	0.8452	0.9504	0.1526	0.072*	
C18	0.7597 (3)	0.91060 (14)	0.2906 (2)	0.0566 (7)	
H18A	0.7298	0.8687	0.2538	0.068*	
C19	0.9524 (4)	1.07473 (16)	0.2168 (3)	0.0979 (12)	
H19A	1.0489	1.0841	0.2614	0.147*	
H19B	0.8950	1.1166	0.2084	0.147*	
H19C	0.9623	1.0575	0.1388	0.147*	

### Atomic displacement parameters (Å^2^)

**Table:**

	*U*^11^	*U*^22^	*U*^33^	*U*^12^	*U*^13^	*U*^23^
P1	0.0361 (3)	0.0466 (4)	0.0374 (3)	0.0011 (4)	0.0089 (2)	−0.0014 (3)
O1	0.0345 (10)	0.0549 (12)	0.0549 (9)	−0.0006 (10)	0.0065 (7)	0.0005 (8)
O2	0.0502 (12)	0.0530 (12)	0.0600 (10)	0.0080 (10)	0.0210 (8)	0.0112 (8)
N1	0.0438 (13)	0.0734 (16)	0.0488 (10)	−0.0117 (12)	0.0196 (9)	−0.0200 (10)
N2	0.0561 (13)	0.0520 (15)	0.0416 (9)	−0.0139 (12)	0.0182 (9)	−0.0067 (9)
C1	0.0433 (14)	0.0487 (18)	0.0403 (11)	−0.0051 (14)	0.0141 (10)	−0.0057 (11)
C2	0.073 (2)	0.066 (2)	0.0506 (14)	0.0140 (18)	0.0063 (13)	0.0064 (14)
C3	0.083 (3)	0.067 (2)	0.084 (2)	0.029 (2)	0.0128 (17)	−0.0021 (16)
C4	0.081 (2)	0.071 (2)	0.0782 (19)	−0.003 (2)	0.0282 (17)	−0.0296 (17)
C5	0.092 (2)	0.088 (3)	0.0452 (14)	0.011 (2)	0.0089 (14)	−0.0147 (15)
C6	0.0702 (18)	0.0572 (18)	0.0452 (12)	0.0103 (17)	0.0056 (12)	−0.0002 (13)
C7	0.0493 (17)	0.0437 (16)	0.0391 (12)	−0.0108 (13)	0.0127 (11)	−0.0037 (11)
C8	0.084 (2)	0.088 (2)	0.0474 (14)	0.028 (2)	0.0033 (13)	−0.0066 (15)
C9	0.085 (2)	0.101 (3)	0.0421 (14)	0.013 (2)	0.0043 (14)	−0.0095 (14)
C10	0.083 (2)	0.100 (3)	0.0533 (16)	−0.006 (2)	0.0253 (15)	−0.0225 (15)
C11	0.092 (3)	0.107 (3)	0.0727 (19)	0.041 (2)	0.0048 (17)	−0.0266 (18)
C12	0.065 (2)	0.094 (2)	0.0522 (15)	0.0257 (19)	−0.0001 (13)	−0.0125 (15)
C13	0.0411 (16)	0.0481 (18)	0.0457 (13)	0.0065 (14)	0.0084 (11)	0.0076 (12)
C14	0.110 (3)	0.058 (2)	0.0485 (15)	−0.009 (2)	0.0244 (16)	−0.0067 (14)
C15	0.109 (3)	0.058 (2)	0.0689 (18)	−0.021 (2)	0.0120 (18)	−0.0091 (15)
C16	0.0531 (19)	0.057 (2)	0.0602 (16)	−0.0057 (17)	0.0047 (13)	0.0123 (15)
C17	0.067 (2)	0.068 (2)	0.0466 (14)	−0.0009 (17)	0.0162 (13)	0.0010 (14)
C18	0.0625 (19)	0.0537 (19)	0.0560 (15)	−0.0107 (16)	0.0168 (13)	−0.0086 (13)
C19	0.102 (3)	0.089 (3)	0.103 (2)	−0.028 (2)	0.018 (2)	0.0294 (19)

### Geometric parameters (Å, °)

**Table:**

P1—O1	1.4670 (17)	C8—C9	1.528 (3)
P1—O2	1.6105 (17)	C8—H8A	0.9700
P1—N1	1.6131 (19)	C8—H8B	0.9700
P1—N2	1.6152 (18)	C9—C10	1.486 (4)
O2—C13	1.394 (3)	C9—H9A	0.9700
N1—C1	1.472 (3)	C9—H9B	0.9700
N1—H1	0.7593	C10—C11	1.503 (4)
N2—C7	1.470 (3)	C10—H10A	0.9700
N2—H2	0.7510	C10—H10B	0.9700
C1—C2	1.496 (3)	C11—C12	1.539 (3)
C1—C6	1.513 (3)	C11—H11A	0.9700
C1—H1A	0.9800	C11—H11B	0.9700
C2—C3	1.517 (3)	C12—H12A	0.9700
C2—H2A	0.9700	C12—H12B	0.9700
C2—H2B	0.9700	C13—C14	1.354 (3)
C3—C4	1.522 (3)	C13—C18	1.364 (3)
C3—H3A	0.9700	C14—C15	1.367 (4)
C3—H3B	0.9700	C14—H14A	0.9300
C4—C5	1.500 (4)	C15—C16	1.362 (3)
C4—H4A	0.9700	C15—H15A	0.9300
C4—H4B	0.9700	C16—C17	1.369 (3)
C5—C6	1.536 (3)	C16—C19	1.505 (4)
C5—H5A	0.9700	C17—C18	1.380 (3)
C5—H5B	0.9700	C17—H17A	0.9300
C6—H6A	0.9700	C18—H18A	0.9300
C6—H6B	0.9700	C19—H19A	0.9600
C7—C12	1.490 (3)	C19—H19B	0.9600
C7—C8	1.506 (3)	C19—H19C	0.9600
C7—H7A	0.9800		
			
O1—P1—O2	108.41 (10)	C7—C8—C9	112.0 (2)
O1—P1—N1	114.19 (10)	C7—C8—H8A	109.2
O2—P1—N1	109.13 (10)	C9—C8—H8A	109.2
O1—P1—N2	118.58 (9)	C7—C8—H8B	109.2
O2—P1—N2	101.48 (10)	C9—C8—H8B	109.2
N1—P1—N2	104.07 (11)	H8A—C8—H8B	107.9
C13—O2—P1	123.52 (15)	C10—C9—C8	111.1 (2)
C1—N1—P1	125.28 (16)	C10—C9—H9A	109.4
C1—N1—H1	115.1	C8—C9—H9A	109.4
P1—N1—H1	113.9	C10—C9—H9B	109.4
C7—N2—P1	123.76 (16)	C8—C9—H9B	109.4
C7—N2—H2	115.8	H9A—C9—H9B	108.0
P1—N2—H2	114.6	C9—C10—C11	110.4 (2)
N1—C1—C2	112.93 (18)	C9—C10—H10A	109.6
N1—C1—C6	109.3 (2)	C11—C10—H10A	109.6
C2—C1—C6	110.6 (2)	C9—C10—H10B	109.6
N1—C1—H1A	107.9	C11—C10—H10B	109.6
C2—C1—H1A	107.9	H10A—C10—H10B	108.1
C6—C1—H1A	107.9	C10—C11—C12	111.1 (3)
C1—C2—C3	112.0 (2)	C10—C11—H11A	109.4
C1—C2—H2A	109.2	C12—C11—H11A	109.4
C3—C2—H2A	109.2	C10—C11—H11B	109.4
C1—C2—H2B	109.2	C12—C11—H11B	109.4
C3—C2—H2B	109.2	H11A—C11—H11B	108.0
H2A—C2—H2B	107.9	C7—C12—C11	112.2 (2)
C2—C3—C4	110.7 (2)	C7—C12—H12A	109.2
C2—C3—H3A	109.5	C11—C12—H12A	109.2
C4—C3—H3A	109.5	C7—C12—H12B	109.2
C2—C3—H3B	109.5	C11—C12—H12B	109.2
C4—C3—H3B	109.5	H12A—C12—H12B	107.9
H3A—C3—H3B	108.1	C14—C13—C18	119.5 (2)
C5—C4—C3	110.1 (2)	C14—C13—O2	118.4 (2)
C5—C4—H4A	109.6	C18—C13—O2	122.0 (2)
C3—C4—H4A	109.6	C13—C14—C15	120.1 (2)
C5—C4—H4B	109.6	C13—C14—H14A	120.0
C3—C4—H4B	109.6	C15—C14—H14A	120.0
H4A—C4—H4B	108.2	C16—C15—C14	122.1 (3)
C4—C5—C6	111.9 (2)	C16—C15—H15A	118.9
C4—C5—H5A	109.2	C14—C15—H15A	118.9
C6—C5—H5A	109.2	C15—C16—C17	117.1 (3)
C4—C5—H5B	109.2	C15—C16—C19	121.4 (3)
C6—C5—H5B	109.2	C17—C16—C19	121.5 (3)
H5A—C5—H5B	107.9	C16—C17—C18	121.6 (2)
C1—C6—C5	110.8 (2)	C16—C17—H17A	119.2
C1—C6—H6A	109.5	C18—C17—H17A	119.2
C5—C6—H6A	109.5	C13—C18—C17	119.6 (2)
C1—C6—H6B	109.5	C13—C18—H18A	120.2
C5—C6—H6B	109.5	C17—C18—H18A	120.2
H6A—C6—H6B	108.1	C16—C19—H19A	109.5
N2—C7—C12	112.36 (19)	C16—C19—H19B	109.5
N2—C7—C8	110.7 (2)	H19A—C19—H19B	109.5
C12—C7—C8	110.73 (19)	C16—C19—H19C	109.5
N2—C7—H7A	107.6	H19A—C19—H19C	109.5
C12—C7—H7A	107.6	H19B—C19—H19C	109.5
C8—C7—H7A	107.6		
			
O1—P1—O2—C13	29.02 (19)	N2—C7—C8—C9	−179.1 (2)
N1—P1—O2—C13	−95.91 (19)	C12—C7—C8—C9	−53.9 (3)
N2—P1—O2—C13	154.63 (17)	C7—C8—C9—C10	56.4 (3)
O1—P1—N1—C1	−11.6 (2)	C8—C9—C10—C11	−57.2 (3)
O2—P1—N1—C1	109.9 (2)	C9—C10—C11—C12	56.4 (3)
N2—P1—N1—C1	−142.37 (19)	N2—C7—C12—C11	177.5 (2)
O1—P1—N2—C7	70.6 (2)	C8—C7—C12—C11	53.2 (3)
O2—P1—N2—C7	−48.0 (2)	C10—C11—C12—C7	−55.1 (4)
N1—P1—N2—C7	−161.28 (18)	P1—O2—C13—C14	−121.7 (2)
P1—N1—C1—C2	94.3 (2)	P1—O2—C13—C18	61.1 (3)
P1—N1—C1—C6	−142.1 (2)	C18—C13—C14—C15	−0.4 (4)
N1—C1—C2—C3	179.3 (2)	O2—C13—C14—C15	−177.7 (3)
C6—C1—C2—C3	56.4 (3)	C13—C14—C15—C16	−0.1 (5)
C1—C2—C3—C4	−57.1 (3)	C14—C15—C16—C17	0.7 (5)
C2—C3—C4—C5	56.1 (3)	C14—C15—C16—C19	−179.6 (3)
C3—C4—C5—C6	−55.9 (3)	C15—C16—C17—C18	−0.9 (4)
N1—C1—C6—C5	−179.8 (2)	C19—C16—C17—C18	179.5 (3)
C2—C1—C6—C5	−54.8 (3)	C14—C13—C18—C17	0.2 (4)
C4—C5—C6—C1	55.7 (3)	O2—C13—C18—C17	177.4 (2)
P1—N2—C7—C12	110.5 (2)	C16—C17—C18—C13	0.4 (4)
P1—N2—C7—C8	−125.2 (2)		

### Hydrogen-bond geometry (Å, °)

**Table:**

*D*—H···*A*	*D*—H	H···*A*	*D*···*A*	*D*—H···*A*
N1—H1···O1^i^	0.76	2.23	2.969 (3)	163.
N2—H2···O1^i^	0.75	2.25	2.975 (3)	162.

Symmetry codes: (i) *x*−1/2, −*y*+3/2, *z*.
